# Analyzing Coronary Artery Disease in Patients with Low CAC Scores by 64-Slice MDCT

**DOI:** 10.1100/2012/907062

**Published:** 2012-06-04

**Authors:** Nan-Han Lu, Lee-Ren Yeh, Tai-Been Chen, Yung-Hui Huang, Chung-Ming Kuo, Hueisch-Jy Ding

**Affiliations:** ^1^Department of Information Engineering, I-Shou University, Kaohsiung City 84001, Taiwan; ^2^Department of Radiology, E-DA Hospital, I-Shou University, Kaohsiung City 82445, Taiwan; ^3^Department of Medical Imaging and Radiological Sciences, I-Shou University, Kaohsiung City 82445, Taiwan

## Abstract

*Purpose*. Coronary artery calcification (CAC) scores are widely used to determine risk for Coronary Artery Disease (CAD). A CAC score does not have the diagnostic accuracy needed for CAD. This work uses a novel efficient approach to predict CAD in patients with low CAC scores. *Materials and Methods*. The study group comprised 86 subjects who underwent a screening health examination, including laboratory testing, CAC scanning, and cardiac angiography by 64-slice multidetector computed tomographic angiography. Eleven physiological variables and three personal parameters were investigated in proposed model. Logistic regression was applied to assess the sensitivity, specificity, and accuracy of when using individual variables and CAC score. Meta-analysis combined physiological and personal parameters by logistic regression. *Results*. The diagnostic sensitivity of the CAC score was 14.3% when the CAC score was ≤30. Sensitivity increased to 57.13% using the proposed model. The statistically significant variables, based on beta values and *P* values, were family history, LDL-c, blood pressure, HDL-c, age, triglyceride, and cholesterol. *Conclusions*. The CAC score has low negative predictive value for CAD. This work applied a novel prediction method that uses patient information, including physiological and society parameters. The proposed method increases the accuracy of CAC score for predicting CAD.

## 1. Introduction

Angiography by multidetector computed tomography (MDCT) has played an important role in clinical assessment of coronary artery disease (CAD) in recent years. Coronary computed tomographic (CT) angiography has high detection sensitivity for CAD and a high negative predictive value for excluding obstructive CAD. Many patients cannot undergo coronary CT angiography due to arrhythmia, renal insufficiency, hyperthyroidism, known allergic reaction to the iodinated contrast medium, and an inability to hold one's breath [1–3]. Coronary calcification is an atherosclerotic marker. The coronary artery calcium (CAC) scores by MDCT angiography are a rapid and noninvasive method for quantifying coronary calcium and generates a restrictive prediction for CAD risk [4–6]. Accuracy of CAD decreases markedly for patient groups with CAC scores <400 or those who cannot undergo CT angiography examination [1]. Blooming artifacts due to severe calcification TRY obscure the entire vessel lumen, which is then not easily resolved. In a patient subgroup without calcification of the coronary artery or with calcification (CAC scores ≤30), the incidence of coronary events remains high.

Therefore, this work conducts a meta-analysis of patient data using physiological and individual parameters to improve CAC score accuracy for early detection of CAD.

## 2. Materials and Methods

This study was approved by the institutional internal review board of E-Da Hospital, Taiwan, and informed consent was not required for this retrospective Health Insurance Portability and Accountability Act (HIPAA) investigation. The study group consisted of 86 consecutive patients, 65 males and 21 females (mean age, 54.03 ± 8.96 standard deviation (STD); range, 33–82 years), who were scheduled to undergo coronary CT angiography for health screening. Patient gender, family medical history, smoking status, body weight, body height, blood pressure (BP), age, glycemia score, cholesterol level, triglyceride level, heart rate, high-density lipoprotein cholesterol (HDL-c), low-density lipoprotein cholesterol (LDL-c), and CAC score from 64-slice MDCT angiographic studies were recorded.

All CAC scores were obtained and coronary CT angiographic studies were performed using a 64-slice MDCT scanner (LightSpeed VCT; GE Healthcare, Milwaukee, WI, USA). Heart rate and blood pressure were recorded for each patient before CT examination. If heart rate exceeded 65 beats/min, *β*-blockers were administered orally to patients to reduce their heart rate. Nitroglycerin spray was administered sublingually immediately prior to each scan. Scanning protocol included acquisition of a low-energy tomogram and a bolus timing scan. All patients underwent unenhanced CT imaging to determine their CAC score. The CAC score was calculated using the Agatston method with software identical to that used in the Multi-Ethnic Study of Atherosclerosis [7]. The MDCT coronary angiography was performed with acquisition of 64 slices at 0.625-mm collimation, 120 kVp, 550 mAs, and 0.35 sec gantry rotation time. All CT data were transferred to an offline workstation (Advantage Windows Workstation 4.2; GE Healthcare, US) and were assessed by one senior radiologist.

### 2.1. Personal Basic and Society Parameters

Two individual parameters, family medical history and smoking status, were recorded as positive or negative. Gender was recorded as female or male. These variables were used to identify the effects of a patient's living environment. That individual health is associated with aging is well known. In this work, patient age was recorded in the analytical dataset. Patient body weight (kg) and height (cm) were recorded to identify their effect on CAD. Body weight can affect health of the coronary artery and cause various diseases [8–10]. However, few studies have investigated the effects of body height on CAD [10].

### 2.2. Coronary Artery Calcium (CAC) Score

**Figure 1:**
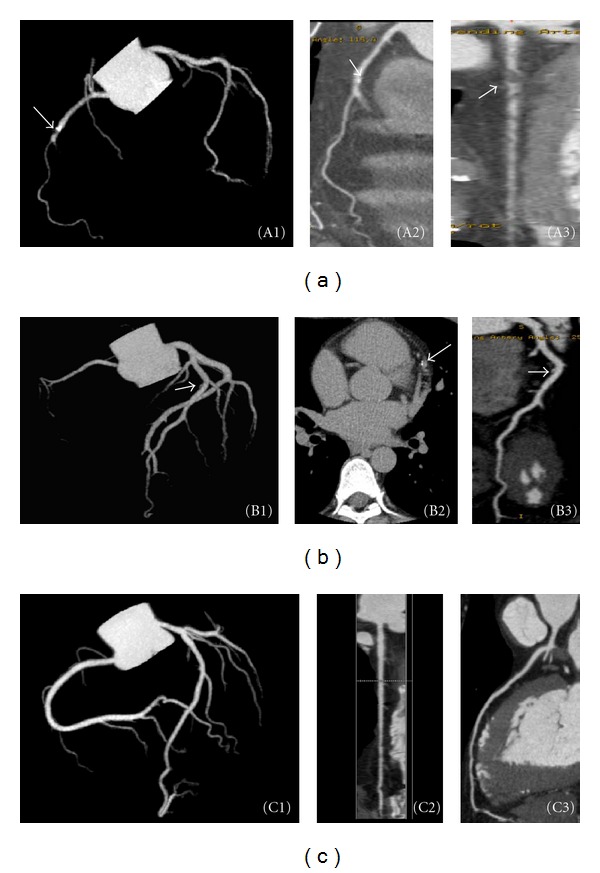
It shows three MDCT images of patients. One 53-year-old male presented with calcified atheroma plaque in the proximal to middle portion of the RCA (A1, A2) and soft plaque in the proximal portion of LAD (A3) and had a CAC score of 110. A 44-year-old male presented with a small amount of calcified atheroma plaque in the proximal to middle portion of the LAD (B1, B2, and B3) and had a CAC score of 5. A 54-year-old male had normal coronary arteries; no calcified atheroma plaque was found (C1, C2, and C3) with his CAC score was 0.

The CAC score was using unenhanced CT images with a detection threshold of 130 HU using semiautomated software (Smart score; GE Healthcare, US). The CAC score, ranging from zero to thousands, reflects the level of CAD. A high CAC score indicates serious CAD. In this work, CAC score was divided into two ranges: 0–30 and >30. For most patient images, the CAC score was in the 0–30 range. In total, 67 of 86 patients had CAC scores of 0–30; that is, there were 53 normal individuals and 14 individuals with CAD.  Figure 1 shows three patient MDCT images. One 53-year-old male presented with calcified atheroma plaque in the proximal to middle portion of the RCA (Figures 1(A1) and 1(A2)) and soft plaque in the proximal portion of the LAD (Figure 1(A3)) with measured CAC score 110. A 44-year-old male presented with calcified atheroma plaque in the proximal to middle portion of the LAD (Figures 1(B1), 1(B2), and 1(B3)) with CAC score 5. A 54-year-old male had normal coronary arteries. No calcified atheroma plaque was found (Figures 1(C1), 1(C2), and 1(C3)) with zero CAC.

### 2.3. Physiological Parameters

Patient BP, triglyceride, cholesterol, blood sugar, heart rate, HDL-c, and LDL-c were important physiological parameters.  Table 1 shows descriptive statistics, including mean, STD, minimum, and maximum values. The parameters of individual parameters, gender, smoking status, and family medical history, also impact CAD. This study evaluates their influence and significance via logistic scores.

### 2.4. Statistical Analysis

Patient data were fitted by logistic regression. Logistic regression is a generalized linear model used for binomial regression and for predicting the probability of an event occurring by fitting data to a logistic curve:

(1) $$\log\;it\left(p_{i}\right)=\ln\left(\frac{p_{i}}{1-p_{i}}\right)=\beta_{0}+\beta_{1}x_{1,i}+\cdots +\beta_{13}x_{13,i},$$

where *p*_*i*_ is the fraction positive for the *i*th abnormal observation, *β*_*i*_ is the *i*th logistic score with respect to *i*th variables, and **β** is used to identify whether the influence a variable is positive or negative.  Table 4 shows the notation for predictors in (1).

**Figure 2:**
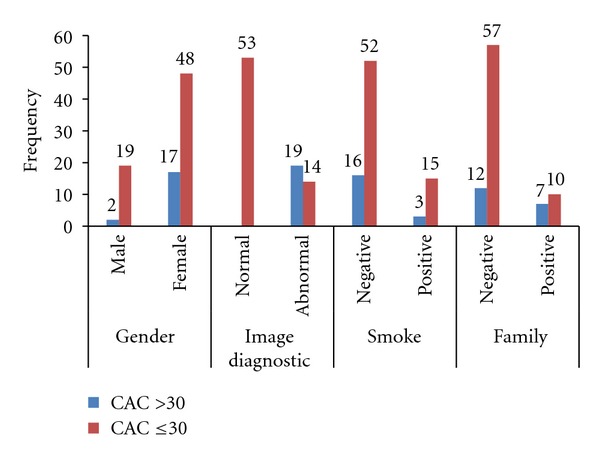
It displays descriptive frequency of categorical variables for the two groups.

Equation (1) determines the diagnostic efficiency of CAC score when the score is ≤30.  Figure 2 shows that 20.89% (14/67) of abnormal cases occurred when the CAC score was ≤30. Conversely, 100% of abnormal cases occurred when the CAC score was >30. Hence, (1) was applied for meta-analysis of selected variables when CAC score was ≤30. Test data were used to evaluate the performance of the logistic model, including its sensitivity, specificity, accuracy, predictive positive value, and predictive negative value. The level of significance (*α*) was 0.05. Statistical analyses were performed using SPSS version 17 for descriptive statistics, logistical model analysis, the receiver operating characteristic (ROC), and area under ROC (AUC).

## 3. Results

**Figure 3:**
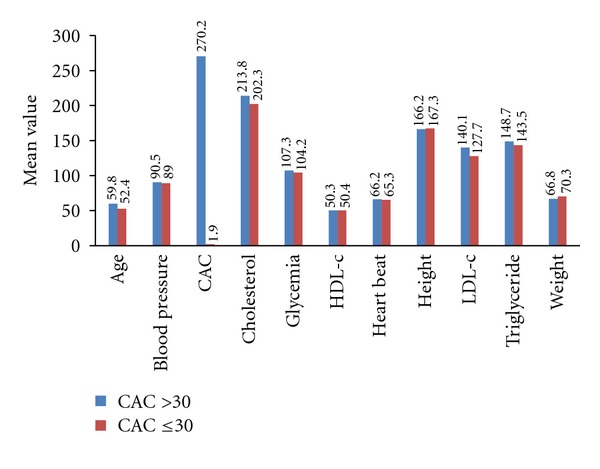
It shows means of predicted variables for the two groups. Mean age, blood pressure, CAC, cholesterol, glycemia, heartbeat, LDL-c, and triglyceride level for the group of CAC scores >30 were larger than those of CAC scores ≤30.

**Figure 4:**
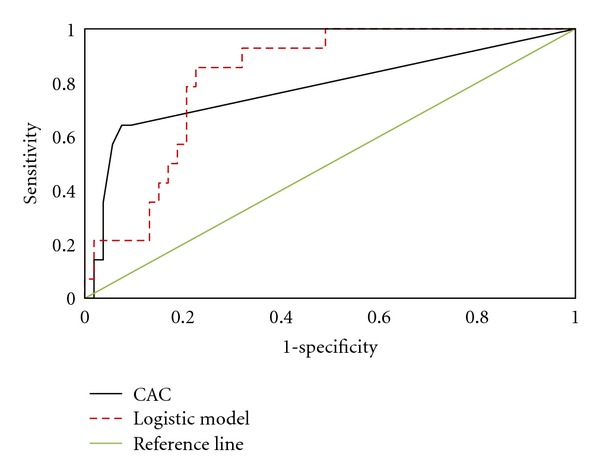
shows ROC curves by (1) and CAC scores ≤30.

Table 1 shows descriptive statistics for variables (are shown for the 86 patients). These data were used to build the logistic model, (1).  Figure 3 shows means of predictive variables for different CAC score ranges. Mean triglyceride level, weight, height, and heart rate for the abnormal group (*n* = 14) (CAC scores ≤30) were larger than those of other groups (*n* = 53).  Table 2 shows the sensitivity, specificity, accuracy, positive predictive value (PPV), negative predictive value (NPV), and area under the ROC curve (AUC) by the logistic model.  Figure 4 shows the ROC curves by (1) for the group with CAC scores ≤30. The proposed model was AUC 84.4%. The proposed model had higher sensitivity, specificity, accuracy, PPV, NPV, and AUC than those by CAC score when CAC score was ≤30. Sensitivity of the CAC score was 100% and 14.30% when the CAC score was >30 and CAC ≤30, respectively, meaning considerable room exists for improving sensitivity using such predictors as blood pressure, age, height, weight, glycemia score, smoking status, family medical history, cholesterol, triglyceride, heart rate, HDL-c, LDL-c, and gender when CAC score is ≤30. Conversely, sensitivity was 71.43% and PPV was 90.90% by meta-analysis via the proposed model for CAC scores ≤30.

**Figure 5:**
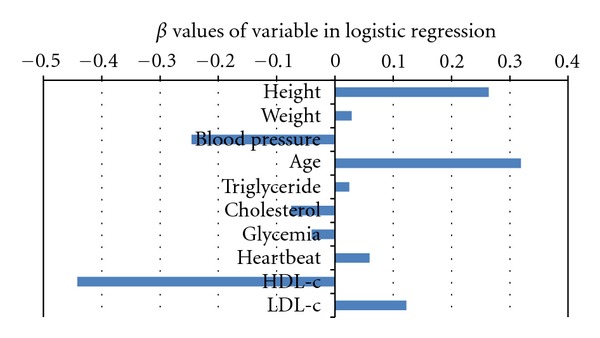
It shows beta scores of variables used in logistic regression. Height, age, blood pressure, HDL-c, and LDL-c are the dominant factors under small CAC scores of ≤30. Height, weight, age, triglyceride, heart rate, and LDL-c positively influenced CAD. The other variables negatively influenced CAD.

Figure 5 shows beta scores of variables used in logistic regression. Height, age, blood pressure, HDL-c, and LDL-c were the dominant factors under CAC scores of ≤30. Height, weight, age, triglyceride, heart rate, and LDL-c positively influenced CAD. The other variables negatively influenced CAD.  Table 3 shows estimated beta values and *P* values of variables using logistic regression for the group with CAC scores <30. Blood pressure, age, triglyceride, cholesterol, HDL-c, LDL-c, and family history were statistically significant (Table 3), meaning that important influenced predictive variables for CAD under CAD scores ≤30.

## 4. Discussion and Conclusions

The CAC score is widely used to diagnose CAD and examine the coronary artery by 64-slice MDCT. In this investigation, the diagnostic sensitivity of CAC score was 14.3% when the CAC score was ≤30. Most patients had CAC scores ranging from zero to hundreds. Useful physiological and individual parameters (variables) are needed to assist in examining the coronary artery or screening for CAD. This work investigated 11 physiological variables—body height, body weight, blood pressure, age, triglyceride, cholesterol, glycemia score, heart rate, HDL-c, LDL-c, and CAC score—and three personal parameters—gender, smoking status, and family medical history. Family medical history, LDL-c, cholesterol, blood pressure, HDL-c, triglyceride, weight, and age improved the diagnostic accuracy of CAC score. The proposed model improved the sensitivity of CAC score by 57.13%. The sensitivity, specificity, accuracy, PPV, NPV, and AUC were >70% after CAC was combined with physiological and personal parameters in the proposed model. The most statistically significant variables were family history, LDL-c, blood pressure, HDL-c, age, triglyceride, and cholesterol according to beta values and *P*-values. 

Body weight was not statistically significant as its range was 47–95 kg in this work. However, body weight was an important factor influencing CAD. Smoking was not statistically significant. Smoking status was recorded as “yes” or “no;” smoking history was not recorded. The weakness of a CAC score when it is ≤30 was identified using logistic models. The important predictive variables were CAC score, family medical history, LDL-c, cholesterol, blood pressure, HDL-c, triglyceride, and age in ascending order based on *P*-values. In the future, identifying quantifiable variables for normal and abnormal cases under the smallest CAC values will prove useful.

In summary, the CAC score has a low negative prediction value for CAD. This work applied a novel meta-analysis method using patient data, including physiological and personal parameters. The proposed method increased the CAC score accuracy in detecting CAD.

## Figures and Tables

**Table 1:** It shows descriptive statistics of variables used in the training model.

Variable	Mean	Std	Min	Max
Blood pressure	89.34	13.14	60	126
Age	54.03	8.96	33	82
Height	167.01	8.21	147	187
Weight	69.52	10.99	47	95
Glycemia	104.88	33.55	75	326
Cholesterol	204.86	32.31	134	279
Triglyceride	144.66	98.34	37	660
CAC score	61.17	188.69	0	1263
Heartbeat	65.51	10.16	42	103
HDL-c	50.35	12.29	29	91
LDL-c	130.45	34.17	54	216

**Table 2:** It shows sensitivity, specificity, accuracy, positive predictive value (PPV), and negative predictive value (NPV), and the AUC for CAC scores ≤30.

Item	Meta-analysis (logistic model)	CAC
Specificity	98.11	98.10
Sensitivity	71.43	14.30
Accuracy	92.54	80.60
Positive predicted value	90.90	33.33
Negative Predicted value	92.86	81.25
AUC	84.40	77.90

**Table 3:** lists variables used in logistic regression and their estimated beta values and *P* values for the group with CAC scores ≤30. Blood pressure, age, triglyceride, cholesterol, HDL-c, LDL-c, and family history are statistically significant.

Variable	Beta	*P* value
Height	0.264	0.087
Weight	0.029	0.779
Blood pressure	−0.246	0.015
Age	0.319	0.021
Triglyceride	0.025	0.026
Cholesterol	−0.075	0.046
Glycemia	−0.040	0.432
Heartbeat	0.060	0.396
HDL-c	−0.442	0.018
LDL-c	0.122	0.014
Gender	24.231	0.996
Smoke	2.176	0.152
Family	6.598	0.011

**Table 4:** It shows the notations for predictors in logistic regression.

Variables	Sign
Diagnostic (positive, negative)	*p*
Blood pressure (Kpa)	*x*_1_
Age (year)	*x*_2_
Height (cm)	*x*_3_
Weight (Kg)	*x*_4_
Triglyceride (*m*mol/L)	*x*_5_
Cholesterol (mg/dL)	*x*_6_
Glycemia (mg/dL)	*x*_7_
Heartbeat (counts per minute)	*x*_8_
High-density lipoprotein cholesterol (mg/dL)	*x*_9_
Low-density lipoprotein cholesterol (mg/dL)	*x*_10_
Gender (male, female)	*x*_11_
Family history (positive, negative)	*x*_12_
Smoke (positive, negative)	*x*_13_
Calcification index	*x*_14_
